# Work competence of general practitioners working in the community health services of Shanghai: a cross-sectional study based on self-assessment

**DOI:** 10.1186/s12909-022-03227-8

**Published:** 2022-03-23

**Authors:** Tianhao Wang, Xueying Ru, Yuan Zhang, Xiangjie Zhang, Jian Gong, Limin Lao, Junling Gao, Zhigang Pan

**Affiliations:** 1 Department of General Practice , Zhongshan Hospital of Fudan University, Shanghai, China; 2 Public Health school, Fudan University, Shanghai, China

**Keywords:** General practitioner, Work competence, Patient care ability, Teaching ability, Communication skill and coordination ability

## Abstract

**Objectives:**

The aim of the study was to investigate the work competence of general practitioners (GPs) in the community health services (CHSs) of Shanghai, China.

**Methods:**

A questionnaire was designed based on a previous capacity evaluation indicator system. We used a stratified and proportional cluster sampling method in this self-assessment and cross-sectional study. We collected data with the questionnaire on GPs’ demographic variables and work competence including patient care ability, teaching ability, communication skill and coordination ability. Univariate analyses were performed by Mann-Whitney U test and Kolmogorov-Smirnov test. Multivariate analyses were done with generalized liner model with significant univariate factors.

**Results:**

A total of 2954 GPs were sampled from 116 CHSs in Shanghai. The response rate was 99.9%. The median scores of patient care ability, teaching ability, communication skill and coordination ability were 80[70–88.75], 76[60–80] and 80[70–85] on a scale of 100, respectively. GPs who were 30–39 years old, or worked in urban CHSs, or took GP trainer’s training or had teaching experience got higher scores in patient care ability. GPs who worked for 5–19 years in CHSs, or worked in CHSs with GP training program or took GP trainer’s training had higher scores in teaching ability. For communication skill and coordination ability, GPs who worked in CHSs with GP standardized training program, or took GP trainer’s training or had teaching experience in CHSs got higher scores.

**Conclusions:**

The work competence of GPs in CHSs of Shanghai could mainly cover daily work, but still needed more improvement in teaching ability.

**Supplementary Information:**

The online version contains supplementary material available at 10.1186/s12909-022-03227-8.

## Introduction

GPs play a crucial role in a new medical model [1] recently introduced in China. Apart from providing medical care services, GPs have taken new challenges as requested in the model including team building and management, communication and teaching in their daily work [2]. Some well-known models, such as the World Organization of National Colleges, Academies and Academic Association of General Practitioners/Family Physicians (WONCA) Tree model [3], the 13 competency model put forward by the Membership of the Royal College of General Practitioners (MRCGP) in England [4], and the Accreditation Council for Graduate Medical Education (ACGME) Program Requirements for Graduate Medical Education in Family Medicine in the United States [5], do not only emphasize medical care, but they also underline the non-clinical skills, like management, leadership, referrals and cooperation with specialists [6, 7]. Nevertheless, due to the lack of a universal model, there was no correct data about the work competence of GPs in China. Even the definition of GP’s work competence is not clear or recognized in China, as far as we know. Some scholars designed questionnaires by Delphi expert consultation and literature review, and carried out empirical studies [8–11]. The indexes in these studies had good reliability and validity, however, some limitations including limited or convenience samples, incomplete contents or less representative objects were the obstacles to generalization. In China, Shanghai is one of the cities where general practice is developed earliest and best, leading the development in China. But the work competence of GPs in Shanghai was rarely studied. Researches on GPs’ work competence are very important for the training and selection of GP talents. In this study, to assess the work competence of GPs in CHSs of Shanghai, China, we conducted a cross-sectional study with a self-designed questionnaire. It is hoped that this study could provide suggestions on the selection of outstanding GP trainers and on the establishment of the work competence model suitable for GPs in the mainland of China.

## Materials and methods

### Study design

We used a stratified and proportional cluster sampling method in the cross-sectional study from Jan, 2017 to Feb, 2017 in CHSs of Shanghai, China.

### Study population and sample

There were 16 districts, 245 CHSs and 5000 GPs in Shanghai in 2016 [12]. 75 CHSs were in seven urban districts and 170 ones in nine rural districts. Sampling was stratified based on the location of CHSs and CHSs with or without GP standardized training program (CHSs with GP standardized training program mean those which were qualified to take the GP standardized training program by Shanghai Health and Family Planning Commission. Until December 2016, a total of 57 CHSs were qualified to take the program in Shanghai). We included all the CHSs with GP standardized training program, and those without were sampled in a ratio of 1 to 1 with those with, in order to get enough eligible responses in the teaching ability survey. Because the number of CHSs with GP standardized training program in each district was different, the number of CHSs without GP standardized training program needed in each district (N) was calculated by the following formula:

$$\mathrm{N}=\mathrm{The}\ \mathrm{number}\ \mathrm{of}\ \mathrm{CHSs}\ \mathrm{without}\ \mathrm{GP}\ \mathrm{standardized}\ \mathrm{training}\ \mathrm{program}\ \mathrm{in}\ \mathrm{each}\ \mathrm{district}/\mathrm{The}\ \mathrm{number}\ \mathrm{of}\ \mathrm{CHSs}\ \mathrm{without}\ \mathrm{GP}\ \mathrm{standardized}\ \mathrm{training}\ \mathrm{program}\ \mathrm{in}\ \mathrm{Shanghai}\times \mathrm{The}\ \mathrm{total}\ \mathrm{number}\ \mathrm{of}\ \mathrm{CHSs}\ \mathrm{without}\ \mathrm{GP}\ \mathrm{standardized}\ \mathrm{training}\ \mathrm{program}\ \mathrm{needed}.$$

### Inclusion criteria and exclusion criteria

All full-time GPs working in these sampled CHSs were invited to complete the questionnaire. GPs who refused this study were excluded.

### Design of questionnaire

The questionnaire was designed on the basis of an indicator system to evaluate primary capacities of GP trainers established by Yuan Zhang et al. [13], which contained 5 parts, including comprehensive performance, capabilities and personal willingness, background of education and career, personal potential for professional development, capacity in general practice, and capacity in training for GPs. There were 57 secondary indexes in the 5 parts, some of which could be confirmed by objective materials. The indexes that could not be verified directly were summed up in this current questionnaire. The indicator system of Yuan Zhang et al. was conducted by two rounds of Delphi expert consultation with 28 related experts. It was assumed that the current questionnaire could evaluate GPs’ work competence well. We performed reliability and validity analyses in this study for the stability and reliability of the results. We classified GPs’ work competence in the current questionnaire as follow: patient care ability, teaching ability, and communication skill and coordination ability. The questionnaire consisted of 4 dimensions:

1. Demographic variables, including gender, age, educational status, professional title, acceptance of GP standardized training (Yes/No), working in CHSs with GP standardized training program (Yes/No), location of CHSs, took GP trainer’s training (Yes/No), and teaching experience (Yes/No).
2. Patient care ability was classified into 3 secondary items with 16 indexes---basic public healthcare, diagnosis and treatment of community common diseases and community clinical skills. This dimension came from parts of the sector of “capacity in general practice” in the initial instrument.
3. Teaching ability mainly showed teaching knowledge and skills, had 10 indexes. This dimension was the necessary indexes in the sector of “capacity in training for GPs” in the initial instrument.
4. Communication skill and coordination ability had 4 indexes. This part was modified from the sector of “comprehensive performance, capacities and personal willingness” in the initial instrument.
5. Each index was evaluated with 5-point Likert scale ranging from 1 to 5, as worse, poor, fair, good and excellent, respectively. The higher the score was, the better the assessment was.

### Data collection

Administrators in each CHS were responsible for distributing and collecting questionnaires. All GPs were encouraged to complete all the questions in their CHSs in 30 min. The answers of all GPs were only used for researches, so we required the GPs to complete them according to their real situations. As the content in the questionnaire was easy to understand, the administrators did not provide any help for GPs and we did not need to train the administrators uniformly. The administrators were also responsible to post the completed questionnaires back to us for which we had paid the postage in advance. Ineligible ones (contradictory or answered less than 50%) were excluded. All scores were converted into percentage system.

### Statistical analysis

First, we conducted the reliability analysis by Cronbach’s coefficient alpha and validity analysis by Kaiser-Meyer-Olkin (KMO) test. We converted two demographic variables, age and work duration, into categorical data and presented them as frequencies and percentage, for the convenience of analyses. Age (y) was divided into 4 groups, < 30, 30 ~ 39, 40 ~ 49, and ≥ 50, and work duration (y) was also divided into 4 groups, < 5, 5 ~ 9, 10 ~ 19, ≥ 20, as in China’s primary care, the four groups of age and work duration always correspond with different professional titles and duties. Continuous data was reported as mean ± SD (standard deviation) if it was on the Gaussian distribution, and as median [IQR (interquartile range)], if not. Univariate analyses were performed by Mann-Whitney U test and Kolmogorov-Smirnov test. Multivariate analyses were done with generalized liner model with significant univariate factors to calculate adjusted odds ratio (OR) and 95% confidence interval (CI). Missing values were filled by multiple imputations if the missing rate was over 10%. The data was analyzed by SPSS Statistics software, version25.0 (SPSS Inc. Chicago). *P* < 0.05 in two-side were considered statistically significant.

## Results

The reliability (Cronbach α) and validity (KMO) of the questionnaire were 0.826 and 0.944.

### Study subjects

**Fig. 1:**
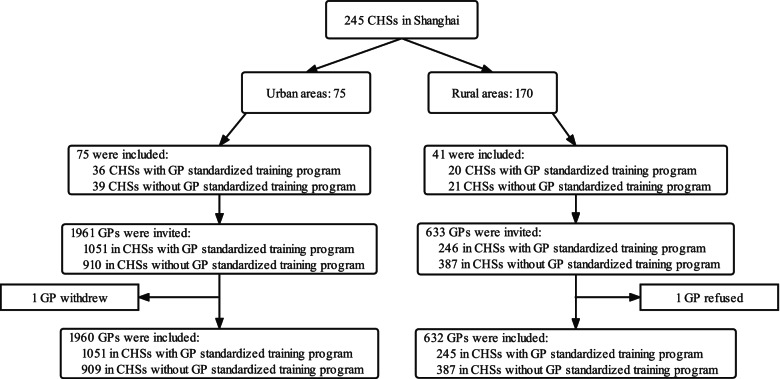
Distribution and flowchart of the sampled CHSs and GPs

The distribution and flowchart of the sampled CHSs and GPs was shown in Fig. 1. 116 CHSs were sampled. The questionnaires were sent to 2594 GPs, and 2592 eligible pieces were returned. The response rate was 99.9%. The demographic data of the 2592 GPs was presented in Table 1. Among the subjects of the study, 1526 (58.9%) were females, whereas 746 (28.8%) were males. Nearly 50% of the subjects were 30–39 years old. 1956 (75.5%) worked in urban CHSs, and 1562 (60.3%) took GP trainer’s training. In addition, 1138 (43.9%) had teaching experience in CHSs.

**Table 1:** Demographic variables of all the 2592 GPs

Variables	N (%)
Num. of CHSs	116 (100.0)
Num. of GPs	2592 (100.0)
Gender	
male	746 (28.8)
female	1526 (58.9)
missing	320 (12.3)
Age(y)	
< 30	186 (7.2)
30–39	1276 (49.2)
40–49	793 (30.6)
≥ 50	286 (11.0)
missing	51 (2.0)
Educational status	
technical secondary school	32 (1.2)
college	265 (10.2)
university	2052 (79.2)
postgraduate and above	241 (9.3)
missing	2 (0.1)
Professional title	
primary	420 (16.2)
intermediate	1848 (71.3)
senior	190 (7.3)
missing	134 (5.2)
Work duration(y)	
< 5	458 (17.7)
5–9	605 (23.3)
10–19	934 (36.0)
≥ 20	583 (22.5)
missing	12 (0.5)
Acceptance of GP standardized training	
yes	1384 (53.4)
no	1204 (46.5)
missing	4 (0.2)
Working in CHSs with GP standardized training program	
yes	1296 (50.0)
no	1296 (50.0)
Location of CHSs	
urban areas	1956 (75.5)
rural areas	628 (24.2)
missing	8 (0.3)
Took GP trainer’s training	
yes	1562 (60.3)
no	1018 (39.3)
missing	12 (0.5)
Teaching experience in CHSs	
yes	1138 (43.9)
no	1417 (54.7)
missing	37 (1.4)

### Score of GPs’ work competence

**Fig. 2:**
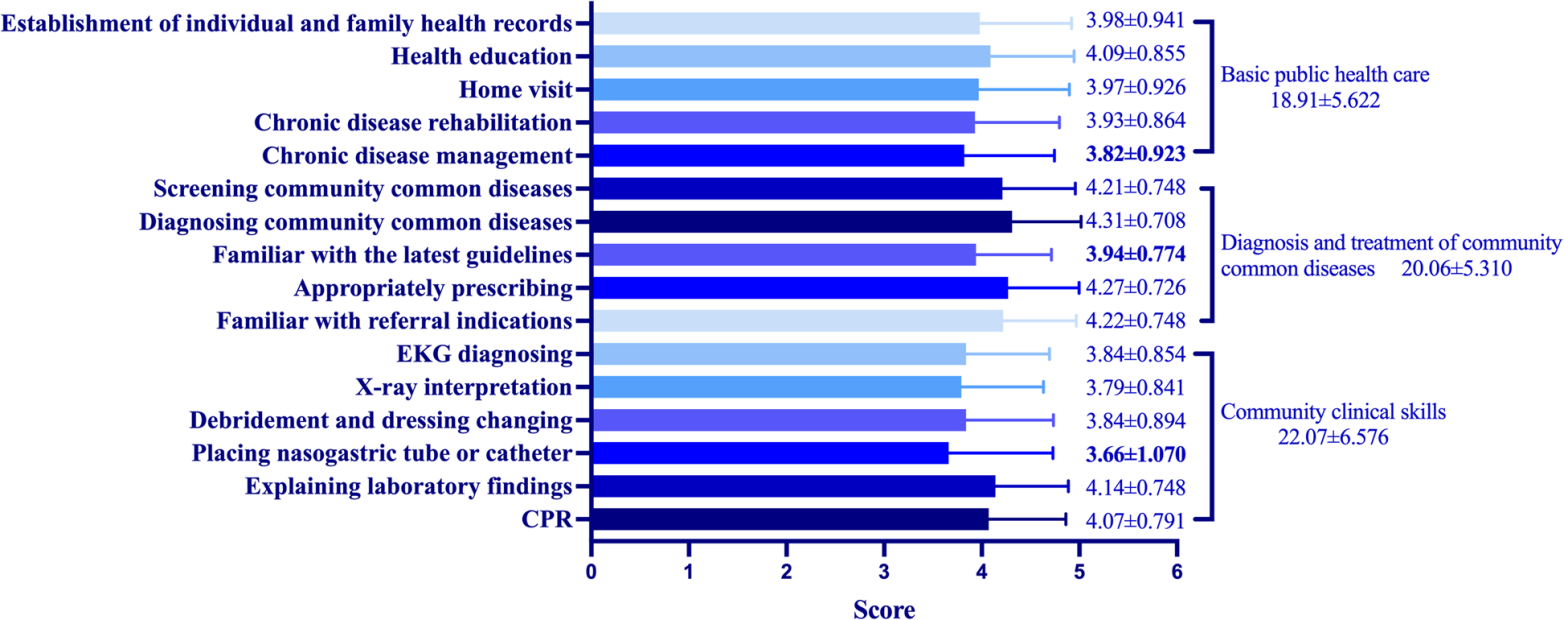
Scores of GPs’ patient care ability

**Fig. 3:**
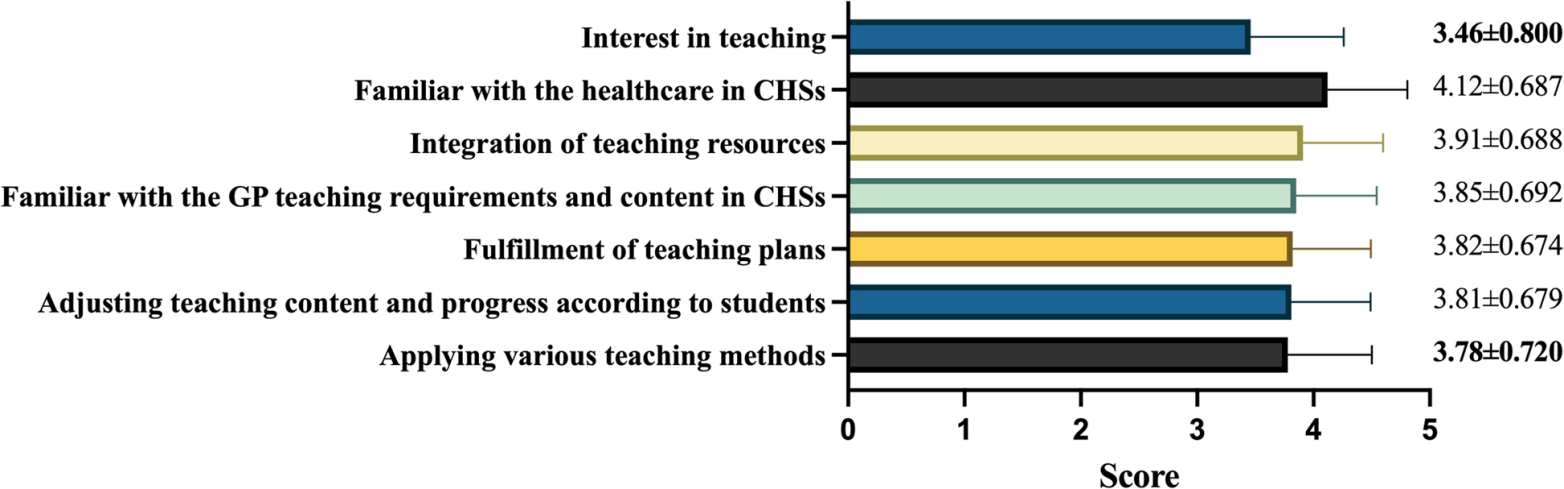
Scores of GPs’ teaching ability

**Fig. 4:**
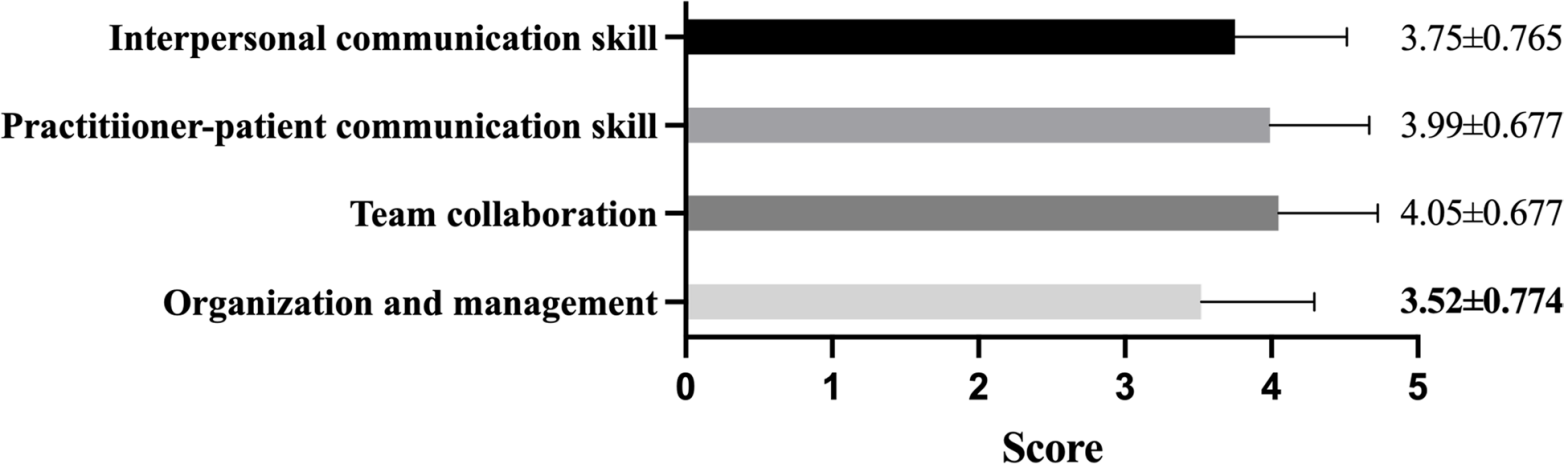
Scores of GPs’ communication skill and coordination ability

The median score of patient care ability was 80 [70–88.75]. The indexes “chronic disease management”, “familiar with the latest guidelines” and “placing nasogastric tube or catheter” had the lowest score in each of the 3 secondary items (basic public healthcare, diagnosis and treatment of community common diseases and community clinical skills) (Fig. 2). The assessment of GPs’ teaching ability was examined among those with teaching experience (*n* = 1138). Median score in this part was 76 [60–80]. Only 75.1% GPs were willing to spend time in teaching. The index “interest in teaching” got the lowest score, 3.46 ± 0.800. The index “applying various teaching methods” was only 3.78 ± 0.720 (Fig. 3). Median score of communication skill and coordination ability was 80 [70–85]. The index “organization and management” had the lowest score, 3.52 ± 0.774. (Fig. 4).

It appeared GPs were confident in their patient care ability, communication skill and coordination ability. Even the score of the three lowest ones in each secondary items of the patient care ability were near 4, which meant “good” as we defined. But the index “organization and management” was relatively weak compared with others. Teaching ability was seemed as the worst, in which the median score was lower than 80, and the index with the lowest score was in this dimension.

### Association between demographic variables and GPs’ work competence

GPs who were 30 to 39 years old [OR = 5.353(1.499 ~ 9.207), *P* = 0.006], or worked in urban CHSs [OR = 2.469(0.351 ~ 4.586), *P* = 0.022], or took GP trainer’s training [OR = 2.761(0.731 ~ 4.808), *P* = 0.008], or had teaching experience [OR = 3.648(1.616 ~ 5.680), *P* < 0.001] got higher scores in the patient care ability. And GPs with GP standardized training got lower scores [OR = -3.004 (− 4.890 ~ − 1.118), *P* = 0.002]. As for teaching ability, GPs who worked for 5 to 19 years [OR = 7.14 (2.468 ~ 11.812), *P* = 0.003 and OR = 5.316(1.302 ~ 9.331), *P* = 0.009], or worked in CHSs with GP standardized training program [OR = 13.226(9.880 ~ 16.572), *P* < 0.001], or took GP trainer’s training [OR = 17.757(13.800 ~ 21.714), *P* < 0.001] got higher scores. For communication skill and coordination ability, GPs who worked in CHSs with GP standardized training program [OR = 1.148(0.066 ~ 2.229), *P* = 0.038], or took GP trainer’s training [OR = 1.484(0.297 ~ 2.671), *P* = 0.014], or had teaching experience in CHSs [OR = 5.174(3.995 ~ 6.354), *P* < 0.001] got higher scores. GPs aged 30–39 years old, or with primary professional titles or GP standardized training got lower scores [OR = -3.578(− 6.229 ~ − 0.928), *P* = 0.008; and OR = -1.858(− 2.951 ~ − 0.766), *P* = 0.001]. (Table 2) After conducting multiple imputation for the “gender” factor, the results were similar to the current ones.

**Table 2:** Association between demographic variables and GPs’ 3 kinds of abilities

Variables	Patient care ability					Teaching ability					Communication skill and coordination ability				
	Score	z/K	*P* value	adjusted OR^c^ (95% CI)	*P* value	Score	z/K	*P* value	adjusted OR^d^ (95% CI)	*P* value	Score	z/K	*P* value	adjusted OR^e^ (95%CI)	*P* value
Gender^a^		−2.413	**0.016**				−1.056	0.291				−2.291	**0.022**		
male	77.24			1.557 (−0.380 ~ 3.494)	0.115	66.95			–	–	77.25			0.866 (−0.253 ~ 1.986)	0.129
female	75.50			0		64.99			–	–	76.23			0	0
Age(y) ^b^		20.588	**< 0.001**				7.489	0.058				21.407	**< 0.001**		
< 30	74.48			2.542 (−3.321 ~ 8.406)	0.395	59.13			–	–	74.73			−0.283 (− 3.496 ~ 2.929)	0.863
30–39	78.12			5.353 (1.499 ~ 9.207)	**0.006**	65.44			–	–	76.31			−2.224 (−4.264 ~ −0.184)	**0.033**
40–49	74.87			2.129 (−1.345 ~ 5.602)	0.23	62.6			–	–	77.89			−1.656 (−3.510 ~ 0.198)	0.080
≥ 50	73.01			0		58.38			–	–	78.91			0	
Educational status^b^		21.359	**< 0.001**				7.192	0.066				7.595	0.055		
technical secondary school	71.09			0.317 (−9.006 ~ 9.640)	0.947	66.57			–	–	74.35			–	–
college	71.50			0.596 (−4.349 ~ 5.542)	0.813	60.17			–	–	76.31			–	–
university	77.02			1.498 (−2.041 ~ 5.036)	0.407	63.65			–	–	77.89			–	–
postgraduate and above	76.69			0		64.83			–	–	78.91			–	–
Professional title†		8.096	**0.017**				4.124	0.127				66.202	**< 0.001**		
primary	75.54			1.598 (−3.037 ~ 6.233)	0.499	59.63			–	–	73.88			−3.578 (−6.229 ~ −0.928)	**0.008**
intermediate	76.54			0.626 (− 3.031 ~ 4.283)	0.737	63.56			–	–	77.46			−1.793 (−3.922 ~ 0.335)	0.099
senior	77.51			0		64.61			–	–	82.08			0	
Work duration(y) ^b^		24.740	**< 0.001**				13.587	**0.004**				27.241	**< 0.001**		
< 5	76.86			3.022 (−0.947 ~ 6.992)	0.136	59.94			2.312 (−3.972 ~ 8.595)	0.471	75.22			1.389 (− 0.843 ~ 3.621)	0.223
5 ~ 9	76.78			−0.072 (−3.384 ~ 3.239)	0.966	65.72			7.14 (2.468 ~ 11.812)	**0.003**	75.71			−0.348 (−2.231 ~ 1.534)	0.717
10 ~ 19	77.73			1.104 (−1.792 ~ 4.000)	0.455	65.61			5.316 (1.302 ~ 9.331)	**0.009**	78.3			0.598 (− 1.058 ~ 2.255)	0.479
≥ 20	73.30			0		58.43			0		77.38			0	
Acceptance of GP standardized training^a^		−1.992	**0.046**				−1.112	0.266				−2.970	**0.003**		
yes	75.33			−3.004 (−4.890 ~ 1.118)	**0.002**	63.09			–	–	76.27			−1.858 (−2.951 ~ −0.766)	**0.001**
no	77.48			0		63.85			–	–	77.66			0	
Working in CHSs with GP standardized training program^a^		−4.357	**< 0.001**				−7.673	**< 0.001**				−5.272	**< 0.001**		
yes	77.65			0.892 (−0.975 ~ 2.758)	0.349	69.24			13.226 (9.880 ~ 16.572)	**< 0.001**	78.32			1.148 (0.066 ~ 2.229)	**0.038**
no	74.97			0		53.44			0		75.54			0	
Location of CHSs^a^		−4.279	**< 0.001**				−3.217	**0.001**				−3.078	**0.002**		
urban area	77.21			2.469 (0.351 ~ 4.586)	**0.022**	64.15			–		77.36			0.058 (−1.168 ~ 1.284)	0.926
rural area	73.55			0		60.48			0		75.6			0	
Took GP trainer’s training^a^		−6.633	**< 0.001**				−7.914	**< 0.001**				−7.431	**< 0.001**		
yes	77.65			2.761 (0.731 ~ 4.808)	**0.008**	67.74			17.757 (13.800 ~ 21.714)	**< 0.001**	78.46			1.484 (0.297 ~ 2.671)	**0.014**
no	74.38			0		47.68			0		74.61			0	
Teaching experience in CHSs^a^		−7.984	**< 0.001**									−12.625	**< 0.001**		
yes	79.00			3.648 (1.616 ~ 5.680)	**< 0.001**	–					80.44			5.174 (3.995 ~ 6.354)	**< 0.001**
no	74.20			0		–					74.06			0	

*Note*: ^a^ Mann-Whitney U test for univariate analysis

^b^ Kolmogorov-Smirnov test for univariate analysis

^c^ adjusted for gender, age, educational status, professional title, work duration, acceptance of GP standardized training or not, working in CHSs with or without GP standardized training program, location of CHSs, took GP trainer’s training or not and teaching experience in CHSs

^d^ adjusted for work duration, working in CHSs with or without GP standardized training program, location of CHSs and took GP trainer’s training or not

^e^ adjusted for gender, age, professional title, work duration, acceptance of GP standardized training or not, working in CHSs with or without GP standardized training program, location of CHSs, took GP trainer’s training or not and teaching experience in CHSs

## Discussion

### Summary

The study, with a self-assessment method, investigated the work competence of GPs in CHSs of Shanghai, China, and suggested that more improvement was needed in teaching ability.

### Strengths and limitations

The study had several strengths. First, this study had the largest sample of 2592 GPs, more than half of the total number of GPs in Shanghai, compared with the published analogous studies in China. With a scientific sample method and a high response rate, the data, to a great extent, had good integrity and representation. Second, though the questionnaire we used was self-designed, we referred to authoritative ones. Thus, the questionnaire had good reliability and validity, and the results and conclusions should be reliable.

There were also some limitations. The major one was the measurement bias and recall bias, because the data we used was collected by GPs’ self-assessment. More objective methods should be adopted in future’s updated assessment. In addition, the conclusion may not be always right outside Shanghai, due to the imbalanced development of general practice in different regions of China.

### Comparison with existing literature

#### Patient care ability

GPs’ median score of patient care ability was 80 [70–88.75]. It was proved that GPs with such ability level could meet the daily working requirements [14]. Some analogous studies in China showed similar results on GPs’ patient care ability [15]. Patient care is GPs’ basic ability [16], and is evaluated first in GP assessments in most countries [17]. It was also one of the reasons why many GPs hoped to attend postgraduation training [18, 19]. In the past GP training, the problem of focusing on theory but ignoring practical skills was common [20]. Xiaoyan Pan et al. [21] discovered that the practice skill score of GPs in Guangxi Province, China was only 63. Such problem was also common among GPs in England and Germany [22–24].

#### Teaching ability

Generally accepted standards were established and used to select eligible GP trainers in many developed countries. In UK, an investigation among specialists on general practice or education showed that to be a qualified GP, 18 competencies were necessary, among which 6 were related to teaching [25]. In General Medical Council (GMC) in 2013, doctors were required that *“you must be competent in all areas of your work, including … teaching”(p.6)” and “You should be prepared to contribute to teaching and training doctors and students”(p.14)”* [26]. Administration as National Health Services (NHS), MRCGP and The Association of Medical Research Charities (AMRC) also reminded GPs of teaching ability [27]. In China, experts recommended that teaching ability was one of the three first-class indicators of the criteria for GP trainers [28].

#### Communication skill and coordination ability

In different health services, communication and coordination ability is a common shortcoming of GPs, though in this study they got not very low scores. Some countries paid attention to the training and assessment of such abilities [29]. For example, the workplace based assessment (WPBA) in MRCGP is aimed to evaluate a doctor’s performance on professional competence across 13 areas in the workplace [30].

### Implications for research and practice

It is urgent and vital to improve GPs’ teaching ability, in order to ensure the effect of GP standardized training. However, lack of eligible trainers was a main problem, similar to our study [31]. Without any formal education on general practice, some GPs in China were far away from being GP trainers [32]. Interest is essential to teaching. This study discovered that nearly 25% GPs were “*unwilling to spend time in teaching*”. GPs are busy in work, and the rewards of teaching was too few to attract GPs’ interest. In training process, students and trainees hoped to get more practical skills, and find the ways of how to offer better healthcare in CHSs. But in most time, the expected effect could not be achieved, partly due to trainers’ failure in using various teaching methods. The cultivation of excellent GPs needs excellent GP trainers [33]. To improve GPs’ teaching ability, what we need to do first is to inspire their interest and establish teaching performance appraisal and reward system [34]. Upper hospitals and medical colleges could set up GP trainer’s training units, hold training lectures regularly [35], and make advanced educational theories and methods permeate CHSs.

Variables related to GPs’ teaching, including taking GP trainer’s training, working in CHSs with GP standardized training program and teaching experience in CHSs, showed positive correlations with the 3 kinds of abilities. This was consistent with the reality. CHSs with GP standardized training program were built in Shanghai since 2012. Compared with those without, CHSs with standardized training program were better in both facility support and GPs’ teaching performance. GP trainer’s training is on-job education to improve teaching ability and generalize advanced methods. Teaching experience also reflected better medical care ability. Excellent GPs would be selected to take trainer’s training on behalf of their CHSs. Only after taking the GP trainer’s training and getting certifications, can they teach students and trainees in their daily work.

What’s more, GPs aged 30 to 39 years old also got higher scores in patient care ability. Many younger GPs were the main force in CHSs, and were much more eager to learn.

However, it was surprising that GPs who accepted GP standardized training got lower scores in patient care ability, and communication skill and coordination ability. GP standardized training is a three-year program. During the 3 years, trainees only spend no more than 1 year in CHSs, which may not be their future work sites. Despite the same work duration, GPs attending such training program might have few experiences of working in CHSs and they are relatively young, so their patient care ability might be unsatisfactory. But there were no related studies, and the exact reasons were not clear. Communication skill and coordination ability is one of the necessary abilities for GPs to get patients’ trust and improve team building and cooperation. Coordination is to solve patients’ health problem in an easiest way in CHSs [36], which was also an important work of GPs. Today, the GP standardized training in China does not pay much attention to communication skill. Young GPs were lacking of management experiences, since leaders of CHSs probably would not assign the management duties to them. So it is suggested to strengthen communication skill training and provide practice opportunities in CHSs in GP standardized training.

## Conclusions

The work competence of GPs in CHSs of Shanghai could mainly cover daily work, but still needed more improvement in teaching ability. Meanwhile, the weaknesses in GP standardized training also need to be made up.

## Supplementary Information

**Additional file 1.** Questionnaire on the work competence of general practitioners in community health services of Shanghai.

## Data Availability

The datasets used and/or analyzed in the current study are available from the corresponding author on reasonable request.

## Abbreviations

ACGME: Accreditation Council for Graduate Medical Education
AMRC: The Association of Medical Research Charities
CHS: Community health service
CI: Confidence interval
GMC: General Medical Council
GP: General practitioner
IQR: Interquartile range
KMO: Kaiser-Meyer-Olkin
MRCGP: Membership of the Royal College of General Practitioners
NHS: National Health Service
OR: Odds ratio
SD: Standard deviation
WONCA: The World Organization of National Colleges, Academies and Academic Association of General Practitioners/Family Physicians
WPBA: The workplace based assessment
