# Supplementary material for: Work competence of general practitioners working in the community health services of Shanghai: a cross-sectional study based on self-assessment
Source: BMC Med Educ. 2022 Mar 23;22:201. doi: 10.1186/s12909-022-03227-8 (PMC8944096; doi:10.1186/s12909-022-03227-8)
Supplement: Supplementary file 1 — Additional file 1. Questionnaire on the work competence of general practitioners in community health services of Shanghai. [file 12909_2022_3227_MOESM1_ESM.docx]

**Code: xxx**

**Questionnaire on the work competence of general practitioners in community health services of Shanghai**

Dear Sir or Madam:

From 2012, Shanghai began the construction of community health services (CHSs) with general practitioner (GP) standardized training program, which was to select eligible CHSs taking responsible for the GP standardized training program. Up till now, 57 CHSs with GP standardized training program were founded. Compared with foreign countries, we have not established any universal models for the assessment of GPs’ work competence. And the work competence of GPs in Shanghai was rarely studied.

Our study aims to investigate the work competence of GPs in CHSs of Shanghai, China.

We guarantee that your answers would be used in nowhere but only in this study. We hope that you complete the questionnaire according to your real thoughts and situations.

Thank you for your attending!

General Department, Zhongshan Hospital

Shanghai Association of Primary Care Providers

Part I: Personal information

1. Name:
2. Gender: □Male □Female
3. Age:
4. Working site:
5. Professional title:
6. Educational status:

□Technical secondary school □College

□University □Postgraduate and above

1. Did you accept and finish the GP standardized training?

□Yes □No

1. Did you take the GP trainer’s training?

□Yes □No

1. Work duration in CHSs? years.

**Introduction:**

Some of the following questions have 5 alternatives on behalf of 5 scales------ worse, poor, fair, good and excellent, ranging from 1 to 5. Please draw “√” in the most appropriate block.

Part II: Primary medical ability

| Secondary index | Third index | 1 | 2 | 3 | 4 | 5 |
| --- | --- | --- | --- | --- | --- | --- |
| Basic Public health care | Establishment of individual and family health records |  |  |  |  |  |
|  | Health education |  |  |  |  |  |
|  | Home visit |  |  |  |  |  |
|  | Chronic disease rehabilitation |  |  |  |  |  |
|  | Chronic disease management |  |  |  |  |  |
| Diagnosis and treatment of community common diseases | Screening community common diseases |  |  |  |  |  |
|  | Diagnosing community common diseases |  |  |  |  |  |
|  | Familiar with the latest guidelines |  |  |  |  |  |
|  | Appropriately prescribing |  |  |  |  |  |
|  | Familiar with referral indicators |  |  |  |  |  |
| Community clinical skills | EKG diagnosing |  |  |  |  |  |
|  | X-ray interpretation |  |  |  |  |  |
|  | Debridement and dressing changing |  |  |  |  |  |
|  | Placing nasogastric tube or urethral |  |  |  |  |  |
|  | Explaining laboratory findings |  |  |  |  |  |
|  | CPR |  |  |  |  |  |

Part II: Teaching ability (only for GPs with teaching experience)

| Index | 1 | 2 | 3 | 4 | 5 |
| --- | --- | --- | --- | --- | --- |
| Interest in teaching |  |  |  |  |  |
| Familiar with the healthcare in CHSs |  |  |  |  |  |
| Integration of teaching resources |  |  |  |  |  |
| Familiar with the GP teaching requirements and content in CHSs |  |  |  |  |  |
| Fulfillment of teaching plans |  |  |  |  |  |
| Adjusting teaching content and progress according to students |  |  |  |  |  |
| Applying various teaching methods |  |  |  |  |  |
| Teaching in clinics |  |  |  |  |  |
| Teaching in ward rounds |  |  |  |  |  |
| Case discussion |  |  |  |  |  |
| Lecture |  |  |  |  |  |

1. Are you willing to spend time in teaching?

□Yes □No

1. Are you willing to take related trainings?

□Yes □No

1. Do you encourage your students in teaching?

□Yes □No

1. Do you give students timely feedback?

□Yes □No

1. Do you evaluate your students monthly?

□Yes □No

Part III: Communication skill and coordination ability

| Index | 1 | 2 | 3 | 4 | 5 |
| --- | --- | --- | --- | --- | --- |
| Interpersonal communication skill |  |  |  |  |  |
| Practitioner-patient communication skill |  |  |  |  |  |
| Team collaboration |  |  |  |  |  |
| Organization and management |  |  |  |  |  |
